# Molecularly Engineered Organic-Inorganic Hybrid Perovskite with Multiple Quantum Well Structure for Multicolored Light-Emitting Diodes

**DOI:** 10.1038/srep33546

**Published:** 2016-09-16

**Authors:** Hongwei Hu, Teddy Salim, Bingbing Chen, Yeng Ming Lam

**Affiliations:** 1 School of Materials Science and Engineering, Nanyang Technological University, 50 Nanyang Avenue, 639798, Singapore

## Abstract

Organic-inorganic hybrid perovskites have the potential to be used as a new class of emitters with tunable emission, high color purity and good ease of fabrication. Recent studies have so far been focused on three-dimensional (3D) perovskites, such as CH_3_NH_3_PbBr_3_ and CH_3_NH_3_PbI_3_ for green and infrared emission. Here, we explore a new series of hybrid perovskite emitters with a general formula of (C_4_H_9_NH_3_)_2_(CH_3_NH_3_)_n−1_Pb_n_I_3n+1_ (where n = 1, 2, 3), which possesses a multiple quantum well structure. The quantum well thickness of these materials is adjustable through simple molecular engineering which results in a continuously tunable bandgap and emission spectra. Deep saturated red emission was obtained with a peak external quantum efficiency of 2.29% and a maximum luminance of 214 cd/m^2^. Green and blue LEDs were also demonstrated through halogen substitutions in these hybrid perovskites. We expect these results to open up the way towards high performance perovskite LEDs through molecular-structure engineering of these perovskite emitters.

Organic-inorganic hybrid perovskite is an interesting class of materials because of their unique molecular structure whereby organic molecules are incorporated into inorganic crystals. In recent years, numerous studies have shown the viability of this material system in functional devices due to their exceptional optical and electrical properties, including high charge mobility, tunable band structure and high luminance efficiency^1^,^2^,^3^,^4^,^5^,^6^,^7^. On top of these functional properties, the solution processability of these hybrid perovskites also enables them to be promising candidates for the next-generation low-cost optoelectronic devices suitable for large-scale production. Since the first introduction of three-dimensional (3D) hybrid perovskites (CH_3_NH_3_PbI_3_ and CH_3_NH_3_PbBr_3_) for photovoltaic applications in 2009, this material system has been seen as a wonder material in the field with a high power conversion efficiency of up to 20% and this is achieved in just a few years^8^,^9^,^10^,^11^,^12^,^13^. This success has also triggered researchers to further explore the application of hybrid perovskites in other areas including light-emitting diode (LED) and laser^14^,^15^,^16^. Recently, room-temperature infrared and green electroluminescence from the 3D hybrid perovskites have been reported confirming their potential for highly efficient, low-cost LED devices^14^. The performance of 3D perovskite LEDs has been dramatically improved through controlled film formation and employing nano-sized crystals to confine the exciton diffusion length^17^,^18^. Perovskite LEDs with a high current efficiency (CE) of 42.9 cd/A were demonstrated very recently, and this once again reinforces the belief that they have strong potential to compete as a new material in light-emission devices^19^.

Despite recent rigorous explorations of 3D perovskites, the first perovskite-based LED demonstrated in 1994 was in fact based on layered hybrid perovskite ((C_6_H_5_C_2_H_4_NH_3_)_2_PbI_4_), which inherently possessed a two-dimensional (2D) quantum well structure^20^. Within those quantum wells, electrons and holes are strongly confined in two dimensions rendering a large exciton binding energy and hence high luminescence yield. Furthermore, multiple quantum well structure, as in the case of conventional inorganic LEDs, has also demonstrated other advantages such as suppression of charge flow and improvement of radiative recombination^21^. However at that time, the efficient electroluminescence from those 2D perovskite LEDs could only be preserved at cryogenic temperature possibly due to exciton quenching at room temperature^20^. Therefore, this poses a huge challenge for practical applications of these materials as light emitting devices.

Extending the pioneering work on both 3D and 2D perovskites, herein we report the first demonstration of room-temperature light-emitting devices based on layered perovskites possessing multiple quantum well (MQW) structure. The quantum well thickness was adjusted by combining the 3D and 2D perovskites at molecular level, resulting in tunable light emission due to the quantum confinement effect. Red perovskite LED with high color purity was obtained with a maximum external quantum efficiency of 2.29% and a maximum luminance of 214 cd/m^2^. Green and blue LEDs based on MQW perovskite were also demonstrated for a solution-processed full-color perovskite display.

## Results

### Perovskite structures and optical properties

**Figure 1:**
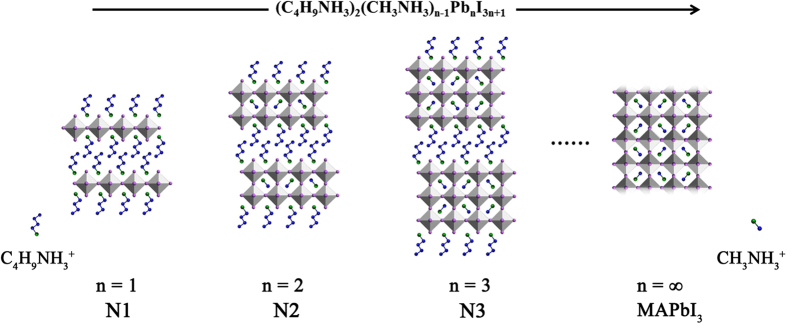
Crystal structures of 2D and 3D perovskites viewed from the side. (C_4_H_9_NH_3_)_2_(CH_3_NH_3_)_n−1_Pb_n_I_3n+1_, where n = 1 for N1, n = 2 for N2, n = 3 for N3 and n = ∞ for MAPbI_3_. The molecular structures of C_4_H_9_NH_3_^+^ and CH_3_NH_3_^+^ are presented with carbon and nitrogen atoms represented by blue and green balls, respectively, while hydrogen atoms are omitted for clarity.

Hybrid perovskites are typically crystallized from the reaction between metal halide and organic halide molecules and subsequently self-organized into 0D, 1D, 2D or 3D structure depending on the connection of metal halide octahedra. For example, the reaction between a small organic molecule such as methylammonium iodide (CH_3_NH_3_I, MAI) and PbI_2_ will result in 3D methylammonium lead iodide (MAPbI_3_) perovskite where the PbI_6_ octahedron connects three-dimensionally with each other by corner-sharing (MAPbI_3_ in Fig. 1). Replacing MAI with larger organic molecules, such as butylammonium iodide (C_4_H_9_NH_3_I, BAI), interrupts the connection in one direction forming a layered structure with inorganic and organic components stacking alternately hence reducing the structural dimensionality (N1 in Fig. 1). In this case, each inorganic octahedra layer is separated by two layers of organic moiety; their interlayer interaction becomes weaker than in the 3D form^22^,^23^,^24^. Therefore, they can be regarded as “bulk 2D” materials comprising of sheet-like inorganic blocks with 2D characteristics. Due to the smaller band gap (E_g_) of the inorganic layers compared to that of the organic layers, quantum wells are formed in these layered perovskites with organic layers acting as barriers confining the inorganic wells^25^. The combination of organic moieties with different sizes (e.g., MA^+^ and BA^+^) allows further fine-tuning of the structure as well as the thickness of the quantum well resulting in double octahedra stacking (N2), triple octahedra stacking (N3), and so on^26^,^27^. The generic chemical formula for these layered hybrid perovskites is (C_4_H_9_NH_3_)_2_(CH_3_NH_3_)_n−1_Pb_n_I_3n+1_, where n represents the number of octahedra layers within each quantum well.

**Figure 2:**
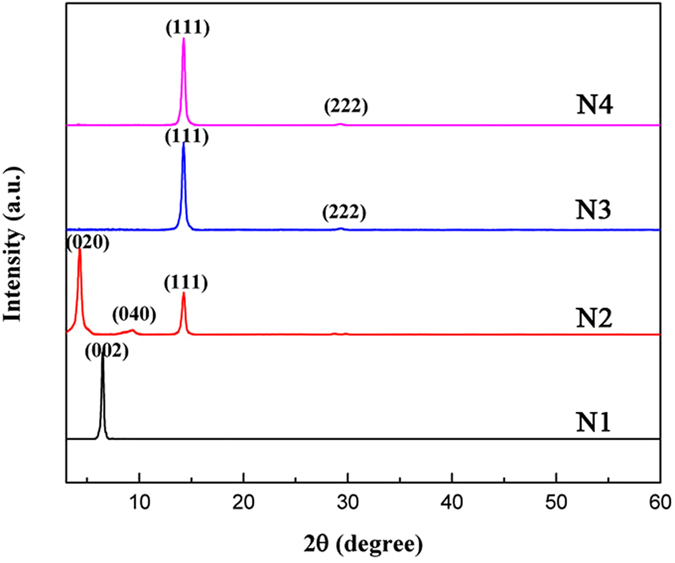
XRD patterns of QW perovskites from N1 to N4.

All the layered perovskite films were spin-coated from solutions containing PbI_2_ and organic halides (BAI for N1, BAI and MAI in respective ratios for N2, N3 and N4). From the change in color of the film, it can be deduced that the film crystallized almost instantaneously - within several seconds after the start of spin coating. The fast crystallization was induced by the self-assembly of both organic molecules and inorganic octahedra which could be advantageous for the proposed quantum well (QW) structure. Essentially, the van der Waals forces between long-chain organic molecules help to drive the organization in these perovskites. The perovskite structure is confirmed by X-ray diffraction (XRD) spectra as shown in Fig. 2. In general, all the layered perovskites showed a tendency to form highly oriented film with crystalline phases comprised of alternate layers of inorganic and organic moieties. For N1, only (002) peak corresponding to the stacking layers, is observed, which indicates high crystallinity and regular layer stacking. The corresponding peak for N2 (020) shifts to a smaller 2θ indicating an enlarged quantum well thickness with wider interplanar spacing. The additional peaks, such as (111) and (222), are due to the inorganic stacking layers and these diffraction peaks become stronger for N3 and N4. It also reveals that growth orientation gradually shifts from (001) to (111) plane with increasing number of octahedra layers (i.e., higher N), as confirmed by previous study^27^. It is worth noting that there is no observable mixed phase between these layered perovskites as confirmed from the high purity of each corresponding XRD pattern. The quantum well and barrier thicknesses can be derived from the XRD patterns as they correspond to the inorganic and organic layer thicknesses respectively. As expected, the QW thickness gradually increases from 0.62 nm for N1 to 2.49 nm for N4 with barrier thickness of around 0.7–0.8 nm. The quantum well and barrier thickness were calculated for the quantum well perovskites and included in the Supplementary Table S1.

**Figure 3:**
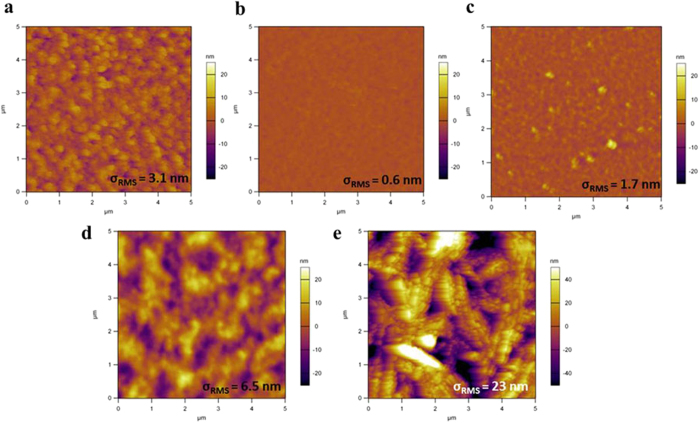
AFM images different perovskite thin films. (**a**) N1, (**b**) N2, (**c**) N3, (**d**) N4 and (**e**) MAPbI_3_ with respective σ_RMS_.

The self-assembly of these hybrid perovskites has a strong influence on the morphology. The N1 perovskite shows large microplates merging into a compact film with a root-mean-square roughness (σ_RMS_) of 3.1 nm (Fig. 3a, Supplementary Fig. S2a). This unique feature derives from the highly ordered stacking layers. Interestingly, for N2 the combination of BAI and MAI results in a smoother film with a σ_RMS_ of 0.6 nm (Fig. 3b, Supplementary Fig. S2b,c). The small roughness of N2 comparable to its single layer thickness suggests a high film uniformity over a large area. The film surface roughness slightly increases for N3 and N4 with σ_RMS_ of 1.7 nm and 6.5 nm, respectively. By contrast, the MAPbI_3_ film is discontinuous and shows a much higher roughness with coverage of about 70% (Fig. 3e, corresponding electron micrograph is shown in Supplementary Fig. S2f). It is clear that the presence of two different organic molecules in the perovskite synthesis has dramatically influenced the film morphology. We believe this is because of the different roles assumed by BAI and MAI during the crystal growth that induce the PbI_6_ octahedra building block to assemble along the plane and c-axis simultaneously. The different sizes of the organic molecules also prevent the mixing of both the organic and inorganic layers resulting in almost perfect assembly of alternating hybrid organic and inorganic layers as confirmed from the distinct XRD peaks.

**Figure 4:**
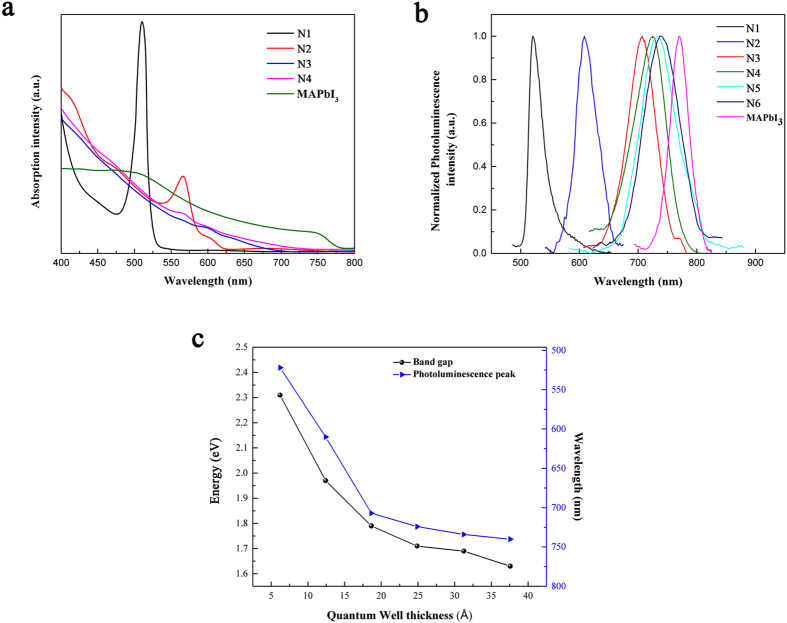
Optical properties of perovskite thin films. (**a**) Optical absorption and (**b**) photoluminescence spectra of different perovskites thin films. (**c**) Optical band gap, photoluminescence peak position and their relationships with the quantum well thickness.

The optical properties of the layered perovskite films were examined and the absorption and luminescence data are shown in Fig. 4. Due to quantum confinement effect, the absorption onset of layered perovskites blue-shifted compared to that of MAPbI_3_. N1 perovskite shows an unusual absorption peak at 511 nm because of the strong photon-exciton interaction confined within the 2D quantum wells. This attributes to the long lifetime and large binding energy (~300 meV) of the excitons^23^. With the increase in the thickness of the quantum wells, this peak becomes weaker and eventually subsides for N3 and N4. The large binding energy is beneficial to promote radiative recombination, as evidenced by the photoluminescence (PL) of the QW perovskites at room temperature. The position of the PL peak (λ_max_) corresponds well to the optical absorption, while the full width at half maximum (FWHM) of the corresponding peak increases from 28 nm for N1 to 74 nm for N6. The correlation between the optical band gap (*E*_g_), λ_max_ and the quantum well thickness is summarized in Fig. 4c. The optical band gap of QW perovskites gradually decreases from 2.3 eV to 1.6 eV approaching the band gap of 3D MAPbI_3_ (~1.55 eV) because of the weaker quantum confinement in the perovskites with thicker quantum wells^28^. The same thickness-dependent trend is also observed for λ_max_ of the PL spectra suggesting that a simple adjustment of the quantum well thickness is able to yield a well-defined emission of the QW perovskites.

### Device performance of quantum well perovskite LEDs

**Figure 5:**
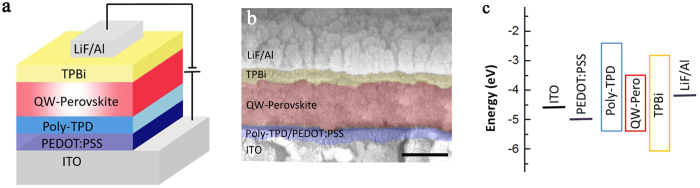
QW perovskite LED structure. (**a**) Schematic of device structure and (**b**) their cross-section scanning electron image, scale bar: 100 nm. (**c**) Energy levels of corresponding layers in QW perovskite LEDs.

To demonstrate their application in light emitting devices, we fabricated QW perovskite LEDs with a multi-layered structure of ITO/poly(ethylenedioxythiophene):polystyrene sulfonate (PEDOT:PSS, 40 nm)/poly(*N,N*′-bis(4-butylphenyl)-*N,N*′-bis(phenyl)-benzidine) (poly-TPD, 30 nm)/QW-perovskite (100 nm)/1,3,5-tris(1-phenyl-1H-benzimidazol-2-yl)benzene (TPBi, 40 nm)/LiF (1 nm)/Al (100 nm), as shown in Fig. 5a,b. Figure 5c shows a schematic of the energy level diagram of all the layers and for the purpose of the diagram, the energy level for QW-perovskite layer is represented by that of N3 obtained from literature^27^. PEDOT:PSS and poly-TPD are solution processed sequentially and they act effectively as the hole-injection layer (HIL) and hole-transport layer (HTL) respectively. Due to its high work function and low electron affinity, the poly-TPD layer reduces the hole-injection barrier from the anode and blocks electrons from the perovskite emitter. A thin layer of TPBi is evaporated on top of perovskite layer to serve as the electron-transporting layer (ETL).

**Figure 6:**
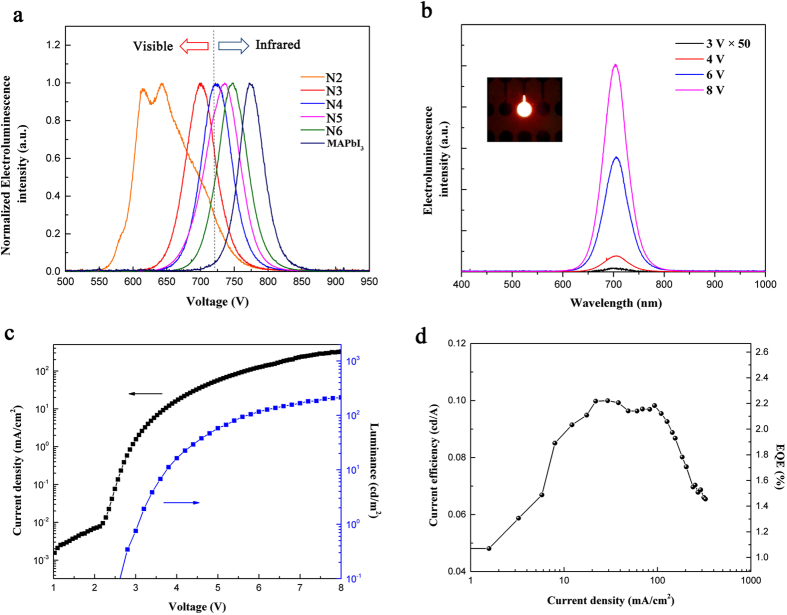
QW perovskite LED characteristics. (**a**) Electroluminescence spectra of QW perovskites and MAPbI_3_. (**b**) Electroluminescence intensity of N3 LED under different voltages; inset shows a N3 LED operated at 6 V. (**c**) *J-V-L* characteristics of N3 perovskite LED. (**d**) Current efficiency and EQE of N3 LED.

No electroluminescence (EL) was observed from N1 based device which is consistent with previous report^20^. However, the remaining layered perovskites in our study (N2–N6) all showed red luminescence and their corresponding EL spectra are shown in Fig. 6a. The N2 perovskite demonstrates an unusual double peak with a broad coverage from 550 nm to 800 nm. The inconsistency between the λ_max_ of EL and PL suggests that the presence of electric field has influenced the electronic structures of N2, which could also explain the absence of EL in N1 device^29^−^32^. The quantum well thickness of N1 and N2 perovskites is only 6.2 Å and 12.4 Å, respectively. Within the narrow quantum wells, the excitons are highly squeezed. The amount of electric field required to drive an LED is enough to cause a massive perturbation on these excitons. Thus the presence of electric field changes their emission characteristics or field-ionizes those excitons, which has been confirmed in the conventional inorganic quantum wells^33^. Further study of the effect of electrical field on the excitons in hybrid multiple quantum well structure is currently being pursued.

For layered perovskites with thicker quantum wells (N3 to N6), their EL spectra correspond well with their respective PL spectra with a narrow peak of FWHM around 50 nm. The dash line in Fig. 6a at 720 nm represents the limit of human visual sensitivity to visible light. Almost the whole emission spectrum of MAPbI_3_ falls into infrared wavelength thus limiting its application in visible displays. By employing quantum well structure, the emission could be gradually shifted into visible wavelength with peaks at 746 nm, 735 nm, 723 nm and 700 nm for N6, N5, N4 and N3, respectively. In particular, the N3 perovskite LED emits deep, saturated red color and 77% of the emitted photons are in the visible spectrum (620 nm to 720 nm). Different operating voltages (3–8V) did not induce any noticeable peak shift in the N3 perovskite LED, as shown in Fig. 6b. The inset shows a small N3 perovskite LED operated at 6 V. It is worth noting that this is the first successful demonstration of a stable pure red emission from perovskite LEDs. Previous study has shown that the red LED fabricated with mixed Br/I perovskite was not stable due to the large discrepancy in the size of both halogens resulting in the film undergoing phase separation under light soaking^34^. Previous approach towards red perovskite LEDs based on Br/I mixed system also resulted in a broad emission spectrum with mixed colors or intermediate orange color^14^,^17^. In our layered perovskite LEDs, pure iodide perovskite was used while the fine tuning of the emission into deep red wavelength region was achieved by adjusting the QW thickness. These devices also yielded highly pure emission with narrow FWHM of 50 nm, which indicates strong quantum confinement effect as well as extremely low amount of impurities in our layered perovskite materials.

The current density-voltage-luminance (*J-V-L*) characteristics of our best LED device based on N3 perovskite are presented in Fig. 6c. The device shows a turn-on voltage (V_on_) of 2.7 V in agreement with the onset of current, suggesting a balanced injection of electrons and holes. The luminance increases steeply after V_on_ and reaches 214 cd/m^2^ at 8 V. The current efficiency (CE) and external quantum efficiency (EQE) increases with current density and yields a maximum CE of 0.1 cd/A and EQE of 2.29% at 21.7 mA/cm^2^ (Fig. 6d). The highest current efficiency could be well maintained (>80%) from 10 mA/cm^2^ to 100 mA/cm^2^, corresponding to a wide range of brightness from 7 cd/m^2^ to 150 cd/m^2^. However, the device shows a dramatic efficiency roll-off at high current density above 100 mA/cm^2^. We speculate that the root cause for this is the increased imbalance between the injection of electrons and holes at higher operating voltage which leads to more non-radiative recombination at the interface. We believe a deeper understanding of device working mechanism as well as the photo-physics of layered perovskite could be beneficial in preventing the efficiency roll-off thus improving its performance.

**Figure 7:**
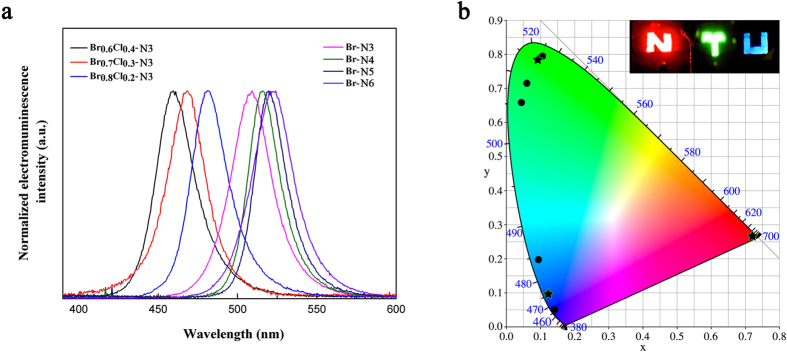
Green and blue LEDs based on QW perovskites. (**a**) Electroluminescence spectra of Br perovskites and Br_x_Cl_1−x_ perovskites. (**b**) CIE coordinates of red, green and blue LEDs based on QW series perovskites; inset shows the photograph of QW perovskite LEDs with Nanyang Technological University (NTU) logo.

For full-color display application, we extend the quantum well structure from iodide-based perovskite to Br and Br/Cl-mixed perovskites to obtain green and blue emission. The same device structure was adopted, i.e., layered perovskite sandwiched between poly-TPD and TPBi. All the layered perovskites exhibit smooth and compact thin films, a strong contrast to the isolated perovskite islands observed in 3D MAPbBr_3_ with a poor coverage of less than 50% (Figure S4). The blue and green EL spectra are shown in Fig. 7a. In the case of bromide-based QW perovskites, similar trend to that of iodide-based perovskites was observed. No emission was detected from Br-N1 and double peak was observed from Br-N2 (see Supplementary Fig. S5). The EL spectra peak (λ_max_) monotonically shifts from 508 nm to 525 nm in the green region for Br-N3 to Br-N6, with a narrow FWHM of ~20 nm. To further drive the emission into the blue region, we mixed a small amount of Cl in Br-N3 and obtained blue emission at 460 nm, 469 nm and 480 nm from Br_0.6_Cl_0.4_-N3, Br_0.7_Cl_0.3_-N3 and Br_0.8_Cl_0.2_-N3, respectively. The device characteristics of our best green and blue LEDs together with the red LEDs are summarized in Table 1. The green LED shows a peak EQE of 1.01%, corresponding to a peak current efficiency of 3.48 cd/A, with a maximum luminance of 2246 cd/m^2^. However, the blue LED has a much lower EQE (0.01%), probably due to the increase in charge injection barrier at both sides which leads to a higher V_on_ of 5.2 V and more quenching of the excitons. We believe the operating voltage as well as the EQE could be improved by replacing the charge injection layers that possess better band alignment with those blue emitting perovskites. As our layered perovskites tend to form uniform films over large area, we fabricated large-area LEDs on 2.5 × 2.5 cm^2^ substrates with patterned electrodes as shown in Fig. 7b. The luminescent logo was constructed with red, green and blue LEDs prepared from I-N3, Br-N5 and Br_0.7_Cl_0.3_-N3 perovskites, respectively. Their high color purity is indicated on the Commission Internationale de l’Eclairage (CIE) diagram by stars while the black circles represent the series of layered perovskites. This demonstrates the promising application for large-area full-color display based solely on hybrid perovskites. The long-term operational stability is a critical concern for hybrid perovskite based photovoltaics and LEDs. Although our layered perovskites show a steady photoluminescence as contrast to the quickly decaying emission for 3D perovskite of MAPb(I_0.5_Br_0.5_)_3_, the device luminescence dropped to ~20% of its initial value after 3 min (see Supplementary Fig. S6). Compared with previous reports where the stability of perovskite LED were only tested under pulsed voltage, our device already showed an improvement mainly due to the smooth, full-coverage perovskite film^14^. Further study to optimize the devices for balanced charge injection and reduced excess current is expected to improve their stability.

## Conclusion

In summary, we fabricated red, green and blue LEDs with hybrid perovskites possessing multiple quantum well (MQW) structure for the first time. These layered perovskites are beneficial in terms of the flexibility in fine-tuning their light emission properties. Furthermore, it also enables the preparation of uniform and smooth film over large area thus making it suitable for scalable manufacturing. We believe that the unique multiple quantum well structure of hybrid perovskite is the way to high-performance light emitting devices. Furthermore, the present study has also opened up a favorable outlook on charge-injection perovskite laser diodes in which multiple quantum wells could be used as optical cavities to amplify light output.

## Methods

### Materials and synthesis

PEDOT:PSS (Clevios PVP Al 4083) was purchased from Heraeus Holding GmbH and used as received. Poly-TPD and TPBi were purchased from American Dye Source and Lumtec, respectively. Other chemicals were purchased from Sigma-Aldrich and used as received. CH_3_NH_3_I (MAI) and C_4_H_9_NH_3_I (BAI) were synthesized from the reaction of methylamine and n-butylamine, respectively, with excess hydriodic acid (HI) (47wt% in water) at 0 °C with (molar ratio of amine to HI = 1:1.2). CH_3_NH_3_Br (MABr) and C_4_H_9_NH_3_Br (BABr) were synthesized similarly with HBr (48wt% in water). The crude product was obtained by slowly evaporating the solvent under reduced pressure. Then the white precipitate was dissolved in ethanol and recrystallized by adding diethyl ether. The small crystals were further washed with diethyl ether several times before drying them in vacuum oven. After drying overnight, they were all sealed under nitrogen and transferred into an N_2_ glove box for further use. (BA)_2_(MA)_n−1_Pb_n_I_3n+1_ precursor solution was prepared by dissolving BAI, MAI and PbI_2_ (99%) with respective stoichiometric ratio in DMF. (BA)_2_(MA)_n−1_Pb_n_Br_3n+1_ precursor solution was prepared similarly using MABr, BABr and PbBr_2_ (99.999%). Mixed Br/Cl perovskite precursor solution was prepared similarly except for the mixing of PbCl_2_ (99.999%) and PbBr_2_ in the solution in a specific ratio. All perovskite precursor solutions were heated at 70 °C before use.

### Device fabrication

To prepare the LED devices, PEDOT:PSS solution was first spin coated onto patterned ITO substrates at 4000 rpm for 60 s, followed by baking at 150 °C for 10 min. After cooling to room temperature, the PEDOT:PSS-coated substrates were transferred into N_2_ filled glove box. Poly-TPD solution (7 mg/ml in chlorobenzene) was spin coated on top of the substrate at 5000 rpm followed by annealing at 110 °C for 30 min. Prior to the perovskite coating, the poly-TPD surface was treated in UV-ozone cleaner for 10 s. The perovskite solution was deposited on the treated substrate at 8000 rpm for 60 s. The perovskite film thickness was controlled by varying the solution concentration. Typically 0.3 M (for Pb^2+^) of N3 perovskite solution results in a film of 100 nm, as examined from scanning electron microscope (SEM) cross section. To obtain comparable thickness, the solution concentrations of N2 and N1 have to be reduced to 0.25 M and 0.2 M, respectively. The LED devices were completed by evaporating TPBi (40 nm), LiF (1 nm) and Al (100 nm) sequentially under high vacuum (1 ×  10^−6^ mbar). The active area was 7 mm^2^ as defined by the overlapping between back electrode and ITO.

### Characterization and device measurement

XRD patterns of the as-deposited perovskite films were collected on Shimadzu XRD-6000 with an incident angle of 1° at 2θ mode. The scan speed was set to 2°/min. SEM and atomic force microscopy (AFM) images were taken on JEOL 7600F and Asylum MFP-3D, respectively. Absorption and photoluminescence spectra were obtained by using Agilent Cary 5000 UV-Vis-NIR and Cary Eclipse Fluorescence Spectrometer. The EL spectra of the LED devices were recorded with Ocean Optics Maya 2000 Pro Spectrometer under working voltages applied by a Keithley 2400 sourcemeter. The current density-voltage-luminance characteristics were collected using a Keithley 2400 sourcemeter, an integrating sphere connected with Maya 2000 Pro Spectrometer through an optical fibre. The corresponding luminance was calibrated with a standard vis-NIR light source (HL-3-INT-CAL, Ocean Optics).

## Additional Information

**How to cite this article**: Hu, H. *et al*. Molecularly Engineered Organic-Inorganic Hybrid Perovskite with Multiple Quantum Well Structure for Multicolored Light-Emitting Diodes. *Sci. Rep.* **6**, 33546; doi: 10.1038/srep33546 (2016).

## Supplementary Material

Supplementary Information

Supplementary Video S1

## Figures and Tables

**Table 1:** Characteristics of red, green and blue perovskite LEDs: emitting perovskite compositions, peak EL wavelength (λ_max_) and FWHM, turn-on voltage (V_on_), peak EQE, peak CE and maximum luminance (L).

Color of Perovskite LEDs	Emitting Perovskite Composition	EL λ_max_ (FWHM)	V_on_	Peak EQE	Peak CE	Max. L
Red	(BA)_2_(MA)_2_Pb_3_I_10_	700 nm (52 nm)	2.7 V	2.29%	0.1 cd/A	214 cd/m^2^
Green	(BA)_2_(MA)_4_Pb_5_Br_16_	523 nm (24 nm)	3.3 V	1.01%	3.48 cd/A	2246 cd/m^2^
Blue	(BA)_2_(MA)_2_Pb_3_Br_7_Cl_3_	468 nm (28 nm)	5.2 V	0.01%	0.006 cd/A	21 cd/m^2^
