# Supplementary material for: Molecularly Engineered Organic-Inorganic Hybrid Perovskite with Multiple Quantum Well Structure for Multicolored Light-Emitting Diodes
Source: Sci Rep. 2016 Sep 16;6:33546. doi: 10.1038/srep33546 (PMC5025709; doi:10.1038/srep33546)
Supplement: Supplementary Information [file srep33546-s1.doc]

**SUPPLEMENTARY INFORMATION**

**Molecular Engineered Organic-Inorganic Hybrid Perovskite with Multiple Quantum Well Structure for Multicolored Light-Emitting Diodes**

Hongwei Hu, Teddy Salim, Bingbing Chen, Yeng Ming Lam^★^

School of Materials Science and Engineering, Nanyang Technological University, 50 Nanyang Avenue, 639798, Singapore.

^★^*E-mail:* [*ymlam@ntu.edu.sg*](mailto:ymlam@ntu.edu.sg)

1. **Quantum Well perovskite structure**

The quantum well (QW) thickness and barrier thickness were derived from XRD results and summarized in Table S1. The spacing d_1_ corresponds to the length of unit cells in c direction which is equal to the total thickness of one pair of quantum well and barrier. The spacing d_2_ indicates the single inorganic octahedra thickness. Therefore the quantum well thickness can be calculated from d_2_ multiplied by the repeating numbers. From N1 to N6, the inorganic octahedra thickness (d_2_) gradually increases indicating the interface stress with organic molecules is released in thicker QWs. The QW thickness of N1 was estimated from the following QW perovskites and the barrier thickness was obtained by subtracting the QW thickness from d_1_.


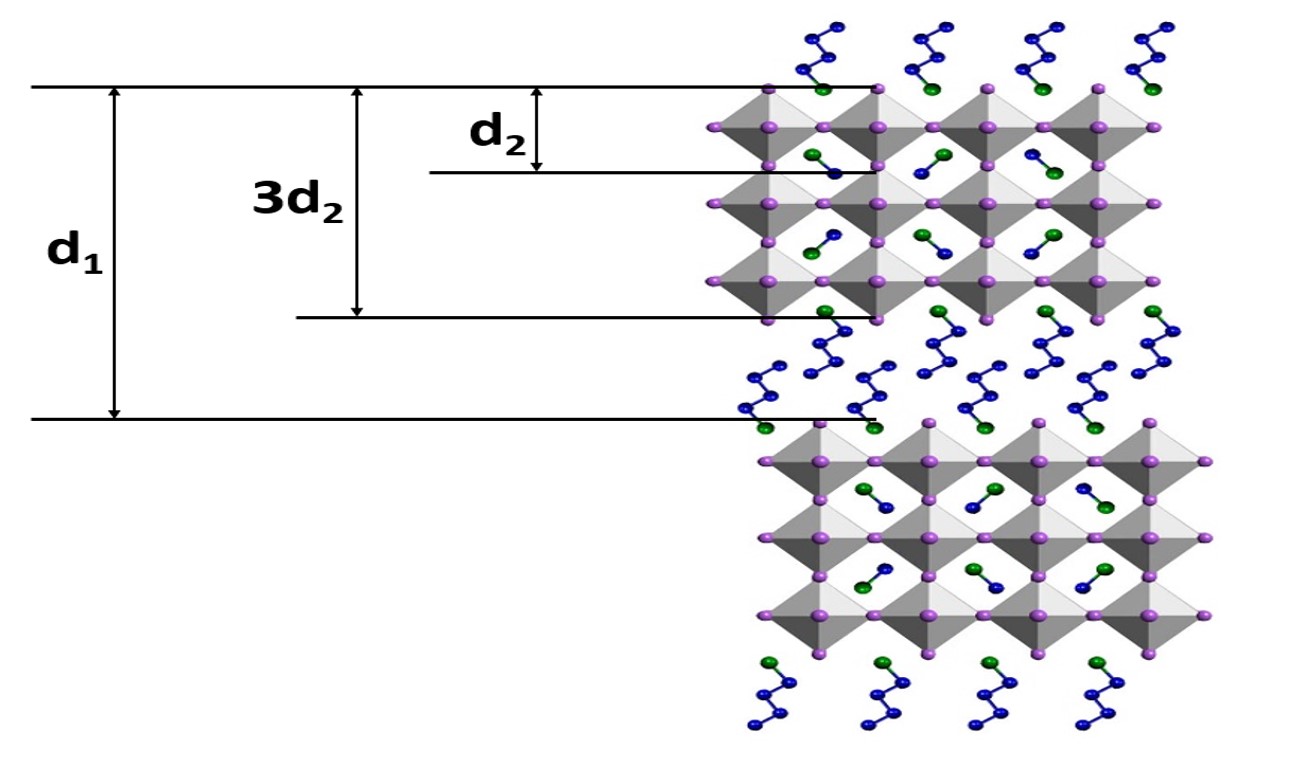


**Figure S1.** Derivation of quantum well and barrier thickness from the crystal structure. d_1_ and d_2_ were obtained from XRD results; the quantum well thickness can be calculated from d_2_ multiplied by the repeating numbers while the barrier thickness was obtained by subtracting the QW thickness from d_1_.

**Table S1** Quantum Well thickness of different QW perovskites derived from XRD patterns

|  | **N1** | **N2** | **N3** | **N4** | **N5** | **N6** |
| --- | --- | --- | --- | --- | --- | --- |
| **d_1_ (‎Å)** | 13.6469 | 20.7149 |  |  |  |  |
| **d_2_ (‎Å)** | ~6.2 | 6.2064 | 6.2121 | 6.2206 | 6.2473 | 6.2599 |
| **Quantum well thickness (‎Å)** | ~6.2 | 12.4128 | 18.6363 | 24.8824 | 31.2365 | 37.5594 |

1. **Surface morphology of perovskites**


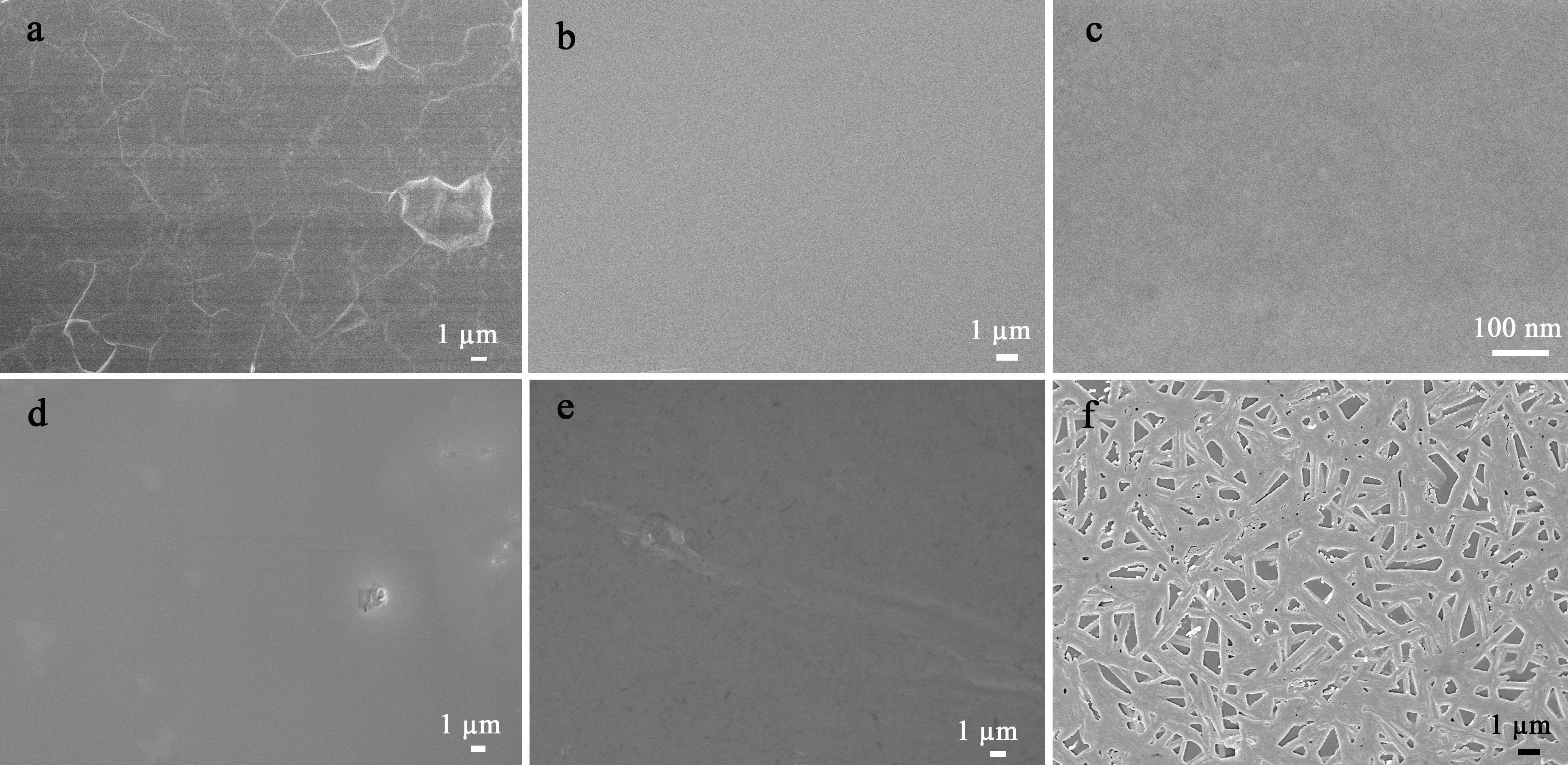


**Figure S2.** Field emission Scanning Electron Microscope (FESEM) images of QW perovskites: N1 (a); low and high resolution images of N2 (b and c); N3 (d); N4 (e) and MAPbI_3_ (f).

1. **Optical band gap of QW perovskites from absorption measurement**


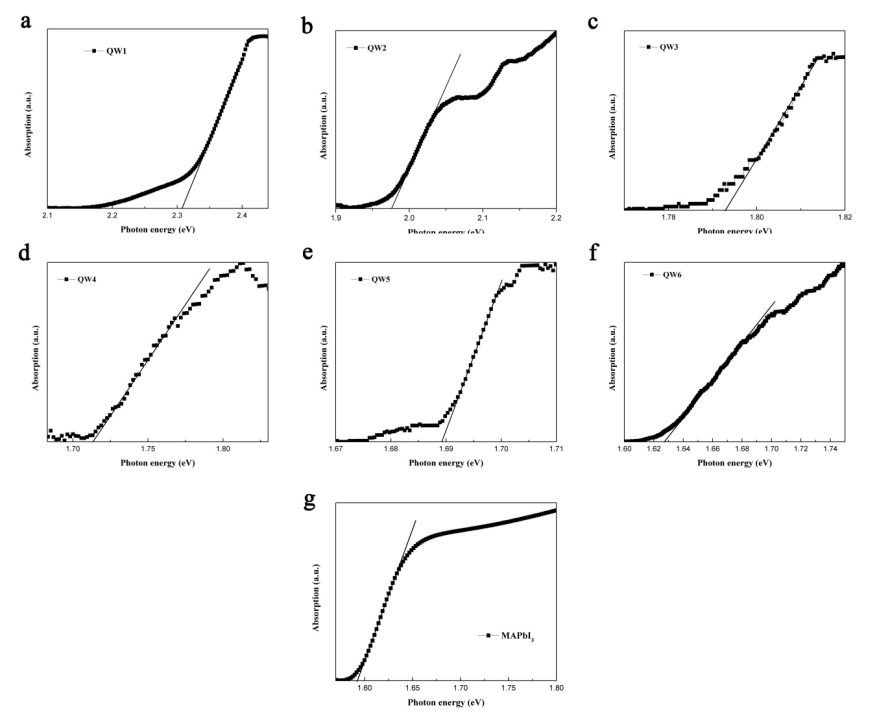


**Figure S3.** Optical band gap of QW perovskites obtained from absorption measurement. (a) N1, (b) N2… (g) MAPbI_3_.

1. **Surface morphology of Br-QW perovskites**


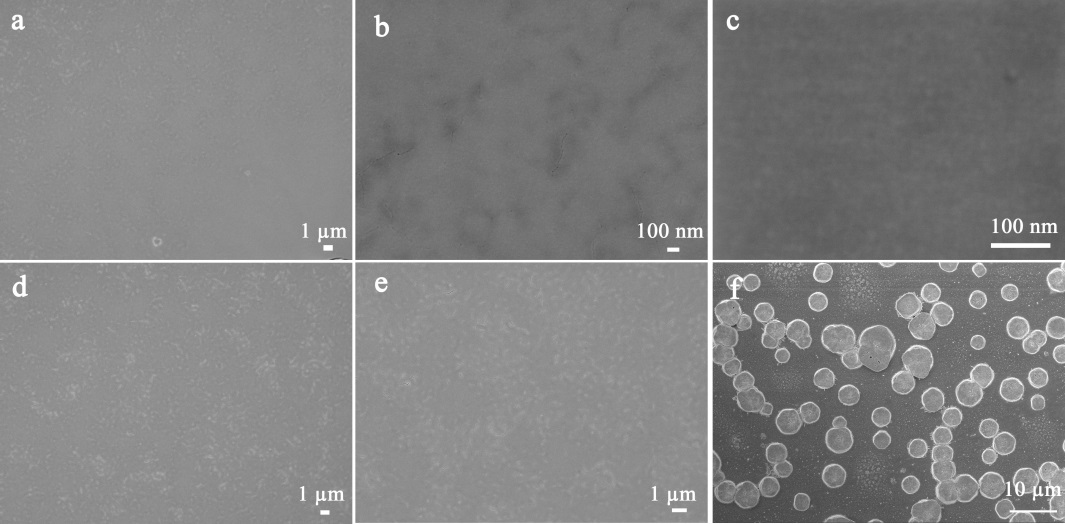


**Figure S4.** Field emission Scanning Electron Microscope (FESEM) images of Br-QW perovskites: low and high resolution images of Br-N2 (a, b and c); Br-N3 (d) and Br-N4 (e); MAPbBr_3_ (f).


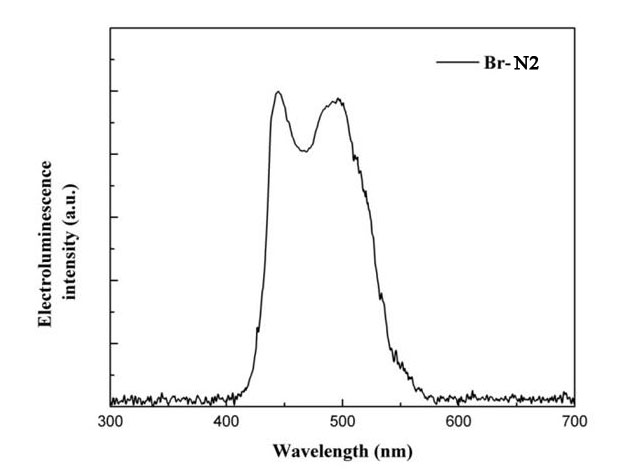


**Figure S5.** Electroluminescence spectra of Br-N2 perovskite LED.

1. **Stability of layered perovskites thin film and device**


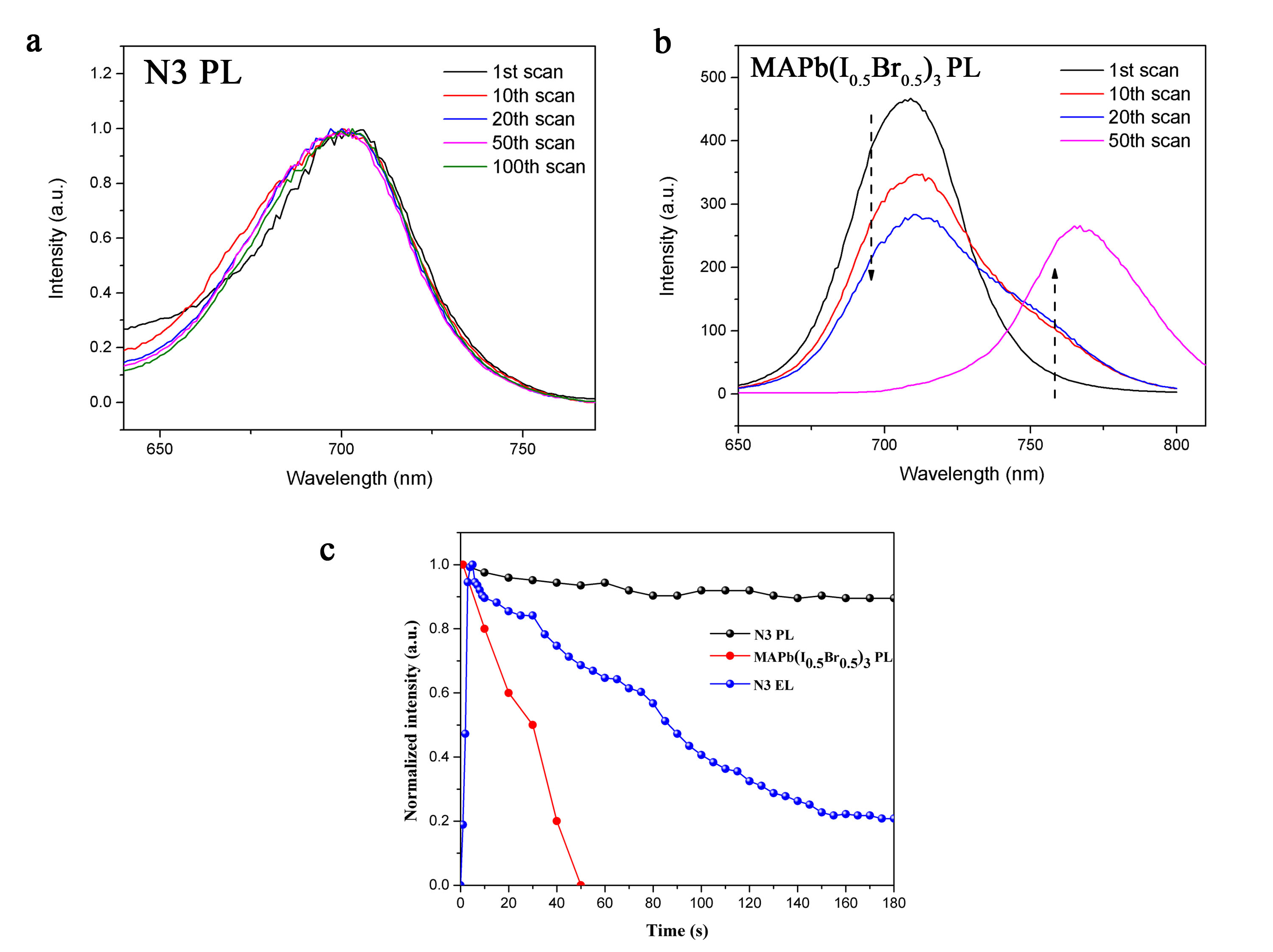


**Figure S6.** Photoluminescence spectra (PL) of N3 and MAPb(I_0.5_Br_0.5_)_3_ under ~ 10 mW/cm^2^ irradiation (a, b). Time dependent luminescence of N3, MAPb(I_0.5_Br_0.5_)_3_ thin film and N3 LED device under 4 V bias (c).

**Supplementary Video Legend**

**Supplementary Video.** A red LED based on N3 perovskite working under constant bias of 4 V.
